# Plan and Scope of an International Dental Congress during World’s Fair, 1903

**Published:** 1901-12

**Authors:** Burton L. Thorpe


### PLAN AND SCOPE OF AN INTERNATIONAL DENTAL CONGRESS DURING WORLD'S FAIR, 1903.

BY DR. BURTON L. THORPE.[1]

THE Louisiana Purchase Exposition will demonstrate to the world the advancement of civilization and the achievements, not only of American citizenship, but of the world's progress.

The best workers in science, art, philosophy, literature, agriculture, trade, and labor will mass and compare the returns of their labors.

American dentistry is recognized the world over for its superior achievements. No profession has made greater strides in scientific research, or attained higher excellence from an artistic or humanitarian stand-point than has our profession. It is, indeed, fitting that dentistry, occupying the high place that it does in science and art, should be represented, as will be all the collateral sciences, in a congress of the progressive dentists of the world, who will come together for professional, scientific, and social purposes, demonstrate the latest methods, and compare results.

The Missouri State Dental Association, believing such a congress would be of great benefit to the profession, has taken initiative steps in this matter by appointing a committee of seven St. Louis dentists, composed of Drs. William Conrad, M. C. Marshall, F. F. Fletcher, Walter M. Bartlett, L. G. McKellops, J. H. Kennerly, and B. L. Thorpe, who have begun the preliminary work, to be followed by the National Dental Association, which will have full charge of the congress.

At the request of Mr. John Schroers, chairman of the Committee on Education for the Louisiana Purchase Exposition, I present the following suggestions as to plan and scope:

The plan followed by the World's Columbian Dental Congress at Chicago in 1893 should be followed, except that a director-general for this congress should be appointed, who would have full charge of all arrangements and devote his whole time to the work of organization.

He should be a man of national reputation, conversant with

---

[1] President Missouri State Dental Association.

details, and able to handle the entire work in a business-like manner. The other officers should be a president, vice-president, secretary-general, and assistant secretaries who possess linguistic abilities, and a treasurer. The main committees should be an Executive Committee, Committee on Finance, Committee on Arrangements, Committee on Invitation, Committee on Printing, Committee on Transportation, Committee on Programme, Committee on Exhibits, Committee on Registration, Committee on Reception, Committee on Education and Literary Exhibits, Committee on Applied Sciences, Committee on Conference, Committee on History, Committee on Nomenclature, Committee on Prize Essays, Committee on Pathology, Committee on Biology and Bacteriology, Committee on Appointment of Dental Surgeons in Armies and Navies of the World, Committee on Care of Teeth of the Poor, besides numerous minor committees. The programme should be presented in sections, each topic classified, thus avoiding confusion, to consist of essays and lantern lectures on anatomy, history, etiology, pathology, bacteriology, chemistry, metallurgy, therapeutics, materia medica, dental and oral surgery, operative and prosthetic dentistry, orthodontia, education, legislation, literature, and clinics by expert operators, to demonstrate the latest methods of operative procedure in dental and oral surgery. The exhibits to consist of biological, bacteriological, and pathological, and the newest improvements in instruments, appliances, and materials. The time between sessions to be devoted to such entertainments as excursions, garden-parties, conversations, receptions, luncheons, and a banquet.

The World's Columbian Dental Congress held at Chicago had a paid membership of one thousand and seventy-four, and was self-sustaining. The St. Louis Congress will likely double the membership of the Chicago Congress.

The following of this plan, with the addition of such features as may be deemed advisable, would give to our profession a great meeting of much benefit to all attending and to the dental world in general.

